# Myxovirus Resistance Protein A as a Marker of Viral Cause of Illness in Children Hospitalized with an Acute Infection

**DOI:** 10.1128/spectrum.02031-21

**Published:** 2022-01-26

**Authors:** Ruut Piri, Mohamed Yahya, Lauri Ivaska, Laura Toivonen, Johanna Lempainen, Kirsi Nuolivirta, Lav Tripathi, Matti Waris, Ville Peltola

**Affiliations:** a Department of Paediatrics and Adolescent Medicine, Turku University Hospital and University of Turku, Turku, Finland; b Institute of Biomedicine, University of Turku, Turku, Finland; c Department of Clinical Microbiology, Turku University Hospital, Turku, Finland; d Department of Pediatrics, Seinäjoki Central Hospital, Seinäjoki, Finland; Memorial Sloan Kettering Cancer Center

**Keywords:** interferon inducible protein, myxovirus resistance protein A, viral infection, bacterial infection

## Abstract

A biomarker for viral infection could improve the differentiation between viral and bacterial infections and reduce antibiotic overuse. We examined blood myxovirus resistance protein A (MxA) as a biomarker for viral infections in children with an acute infection. We recruited 251 children presenting with a clinical suspicion of serious bacterial infection, determined by need for a blood bacterial culture collection, and 14 children with suspected viral infection at two pediatric emergency departments. All children were aged between 4 weeks and 16 years. We classified cases according to the viral, bacterial, or other etiology of the final diagnosis. The ability of MxA to differentiate between viral and bacterial infections was assessed. The median blood MxA levels were 467 (interquartile range, 235 to 812) μg/L in 39 children with a viral infection, 469 (178 to 827) μg/L in 103 children with viral-bacterial coinfection, 119 (68 to 227) μg/L in 75 children with bacterial infection, and 150 (101 to 212) μg/L in 26 children with bacterial infection and coincidental virus finding (*P < *0.001). In a receiver operating characteristics analysis, MxA cutoff level of 256 μg/L differentiated between children with viral and bacterial infections with an area under the curve of 0.81 (95% confidence interval [CI] = 0.73 to 0.90), a sensitivity of 74.4%, and a specificity of 80.0%. In conclusion, MxA protein showed moderate accuracy as a biomarker for symptomatic viral infections in children hospitalized with an acute infection. High prevalence of viral-bacterial coinfections supports the use of MxA in combination with biomarkers of bacterial infection.

**IMPORTANCE** Due to the diagnostic uncertainty concerning the differentiation between viral and bacterial infections, children with viral infections are often treated with antibiotics, predisposing them to adverse effects and contributing to the emerging antibiotic resistance. Since currently available biomarkers only estimate the risk of bacterial infection, a biomarker for viral infection is needed in attempts of reducing antibiotic overuse. Blood MxA protein, which has broad antiviral activity and is rapidly induced in acute, symptomatic viral infections, is a potential biomarker for viral infection. In this diagnostic study of 265 children hospitalized because of an acute infection, blood MxA cutoff level of 256 μg/L discriminated between viral and bacterial infections with a sensitivity of 74% and specificity of 80%. MxA could improve the differential diagnostics of febrile children at the emergency department but, because of frequently detected viral-bacterial coinfections, a combination with biomarkers of bacterial infection may be needed.

## INTRODUCTION

Acute infectious diseases are the most common causes of pediatric emergency department (ED) visits (1, 2). In febrile children at the ED, the reported probabilities of serious bacterial infection vary from 7 to 16% (3–6). Identifying children who require antibiotic treatment remains a major diagnostic challenge. Currently used biomarkers, such as white blood cell count (WBC), plasma C-reactive protein (CRP), and procalcitonin (PCT), have insufficient ability to differentiate between bacterial and viral infections (4–11). Furthermore, highly sensitive multiplex PCR assays for viruses or bacteria, which are routinely used at many EDs, cannot distinguish between an asymptomatic and true infection (12–14). Due to the diagnostic uncertainty, children with viral infections are often treated with antibiotics, predisposing them to adverse effects and contributing to the emerging antibiotic resistance.

Since currently available biomarkers only estimate the risk of bacterial infection, a marker for viral infection is needed. Myxovirus resistance protein A (MxA) is an interferon-inducible protein with broad antiviral activity (15, 16). MxA has potential for use as a biomarker because of its rapid induction in acute, symptomatic viral infections and low levels in bacterial infections and in healthy individuals (17–20). Asymptomatic children positive for a respiratory virus have low MxA levels (21). We have previously reported elevated blood MxA levels in children with acute pharyngitis with both respiratory virus and group A Streptococcus infections but not in children with only group A Streptococcus infection (22). However, MxA response has not been systematically studied in children with coinciding viral and bacterial infections.

The aim of this study was to investigate blood MxA protein as a biomarker for viral infections in children hospitalized with a clinical suspicion of serious bacterial infection.

## RESULTS

### Study population.

**FIG 1:**
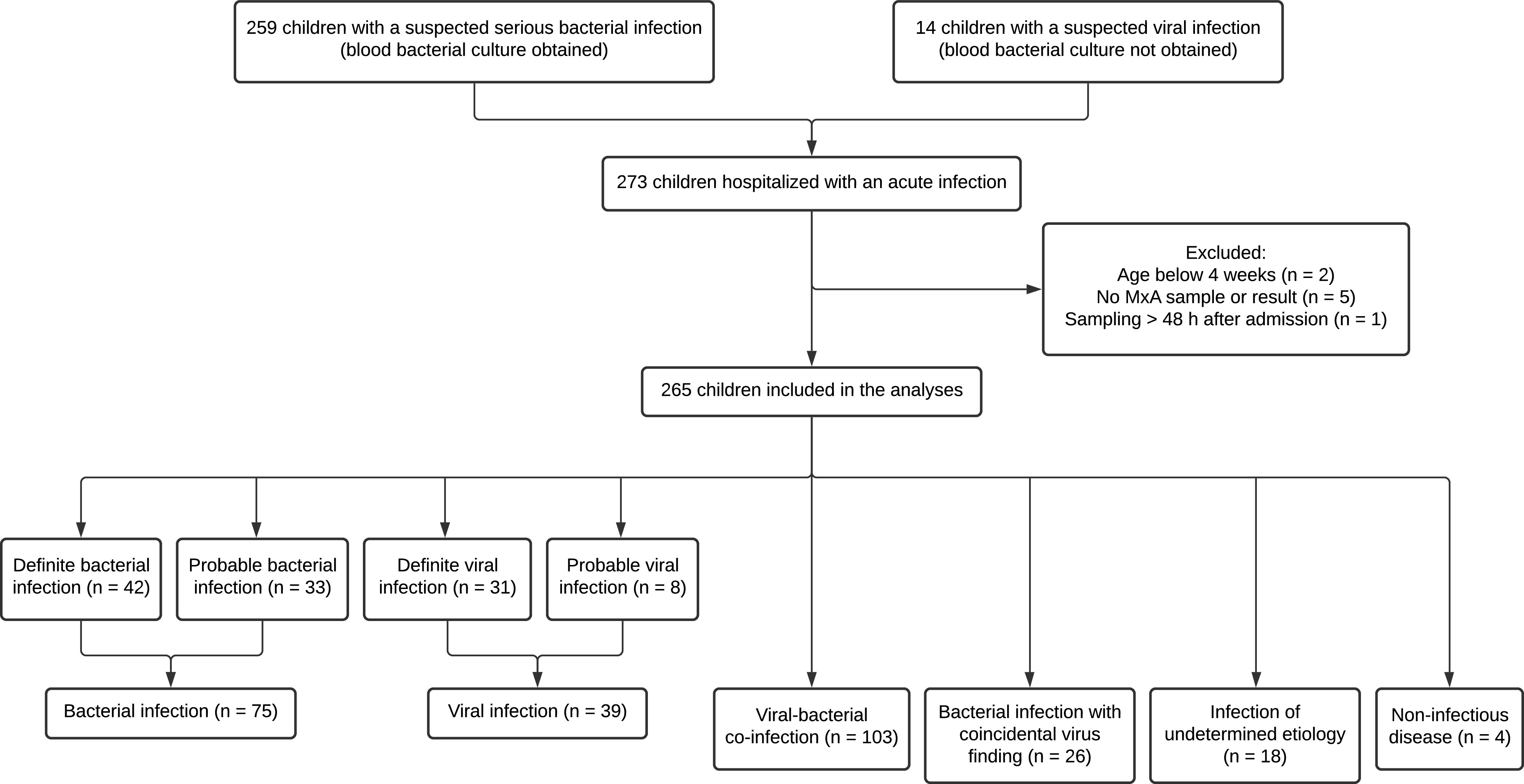
Participant flowchart.

We recruited 259 of 555 eligible children with a suspected serious bacterial infection and a convenience sample of 14 children with suspected viral infection. Two children were excluded because of age less than 4 weeks, five were excluded because of missing blood MxA sample or result, and one child was excluded because of sampling more than 48 h after admission, resulting in a total number of 265 children in the analyses (Fig. 1).

Clinical characteristics, diagnoses, and detected viruses and bacteria are presented in Table 1. The median age of children included in the study was 3.5 years (interquartile range [IQR], 1.3 to 8.1 years). Seven (2.6%) children had an immunosuppressive disease or medication, and 59 (22.3%) had a chronic condition without significant immunosuppression. Pneumonia, pyelonephritis, and skin or soft tissue infections were the most common diagnoses, but viral respiratory infections and generalized viral infections were also frequent definitive diagnoses in children presenting to the ED with a suspected bacterial infection. Children enrolled based on suspected viral infection had most often upper or lower respiratory tract infection or viral gastroenteritis.

**TABLE 1:** Clinical characteristics, diagnoses, and detected viruses and bacteria in 265 study children

Characteristic, diagnosis, or microbe	No. (%) of children
Age	
1–2 mo	28 (10.6)
3–11 mo	29 (10.9)
1–2 yr	65 (24.5)
3–6 yr	61 (23.0)
7–15 yr	82 (30.9)
Sex	
Female	139 (52.5)
Male	126 (47.5)
Chronic conditions	
None	199 (75.1)
Immunosuppressive disease or medication^*a*^	7 (2.6)
Other condition^*b*^	59 (22.3)
Disease characteristics	
Febrile (≥38.0°C) before admission	230 (86.8)
Antibiotic treatment during hospitalization	234 (88.3)
Admitted to intensive care unit	15 (5.7)
Clinical diagnoses	
Pneumonia	81 (30.6)
Pyelonephritis	49 (18.5)
Skin or soft tissue infection	33 (12.5)
Viral respiratory infection^*c*^	22 (8.3)
Tonsillitis	19 (7.2)
Sepsis or toxic shock syndrome	12 (4.5)
Central nervous system infection	8 (3.0)
Chickenpox, herpes zoster, mononucleosis, or enteroviral disease	8 (3.0)
Gastroenteritis	7 (2.6)
Osteomyelitis	5 (1.9)
Virus infection of undetermined etiology	8 (3.0)
Infectious disease of other or undetermined etiology	9 (3.4)
Noninfectious disease^*d*^	4 (1.5)
Respiratory viruses^*e*^	
Rhinovirus	78 (31.5)
Respiratory syncytial virus A or B	27 (10.9)
Human bocavirus	20 (8.1)
Adenovirus	15 (6.0)
Human metapneumovirus	12 (4.8)
Parainfluenza virus 1, 2, 3, or 4	10 (4.0)
Influenza virus A or B	11 (4.4)
Coronavirus V229E, NL63, OC43, or HKU1	11 (4.4)
Enterovirus	5 (2.0)
Any respiratory virus	150 (60.5)
Two or more viruses	34 (13.7)
Other viruses	
Herpesviruses^*f*^	10 (3.8)
Rotavirus	3 (1.1)
Bacterial species isolated from blood or other sterile site^*g*^	
Streptococcus pneumoniae	6 (2.7)
Staphylococcus aureus	6 (2.7)
Escherichia coli	2 (0.8)
Haemophilus influenzae	2 (0.8)
Streptococcus intermedius	1 (0.4)
Salmonella Paratyphi	1 (0.4)

a Juvenile arthritis (*n* = 2), severe combined immunodeficiency, cartilage-hair hypoplasia, liver transplant, sickle cell disease, or total lectin pathway deficiency (*n* = 1 for each).

b Urological or renal disorder (*n* = 12), neurological disorder or syndrome (*n* = 12), asthma (*n* = 10), gastrointestinal disorder (*n* = 5), cardiovascular disease (*n* = 4), endocrine disorder (*n* = 4), hematologic disorder (*n* = 3), birth at <32 weeks (*n* = 3), or other (*n* = 6).

c Upper respiratory tract infection, wheezy bronchitis, laryngitis, or influenza, with or without otitis media or other localized bacterial complication.

d Henoch-Schonlein purpura, Kawasaki disease, or sickle cell crisis.

e Of 248 children studied for respiratory viruses by multiplex PCR.

f Herpes simplex virus, varicella-zoster virus, Epstein-Barr virus, or human herpesvirus 7.

g Cerebrospinal fluid, pleural fluid, or lymph node biopsy.

A respiratory virus was detected in a nasopharyngeal sample in 150 (60.5%) children of 248 studied. Rhinovirus was the most frequent finding, detected in 78 (31.5%) children, followed by respiratory syncytial virus in 27 (10.9%), human bocavirus in 20 (8.1%), and adenovirus in 15 (6.0%). Multiple respiratory viruses were detected in 34 (13.7%) children.

### Etiologic distribution.

We determined 42 (15.8%) of 265 children to have a definite bacterial infection, 33 (12.5%) a probable bacterial infection, 31 (11.7%) a definite viral infection, and 8 (3.0%) a probable viral infection (Fig. 1). There were 103 (38.9%) children with a viral-bacterial coinfection, including those with simultaneous viral and bacterial infections at separate sites, and 26 (9.8%) children with a bacterial infection and coincidental virus finding, of whom 25 were positive for a respiratory virus without respiratory symptoms. Eighteen (6.8%) children had an infection of undetermined etiology, and four (1.5%) had a noninfectious disease. The age distribution and clinical diagnoses for each etiologic group are shown in Tables S1 and S2 in the supplemental material. By combining groups with definite or probable etiology, there were 75 (28.3%) children with a bacterial infection only and 39 (14.7%) with viral infection only. Combining groups with viral infection and viral-bacterial coinfection, there were 142 (53.6%) children with and 105 (39.6%) children without a symptomatic viral infection, when infections of undetermined etiology were excluded. Of 18 children with an infection of undetermined etiology, 10 (55.6%) had a diagnosis of tonsillitis negative for group A Streptococcus, and 7 (39%) tested positive for a respiratory virus but could not be categorized as viral infection only, most often due to an unusually high CRP level.

### Blood MxA protein as a marker of symptomatic viral infection.

**FIG 2:**
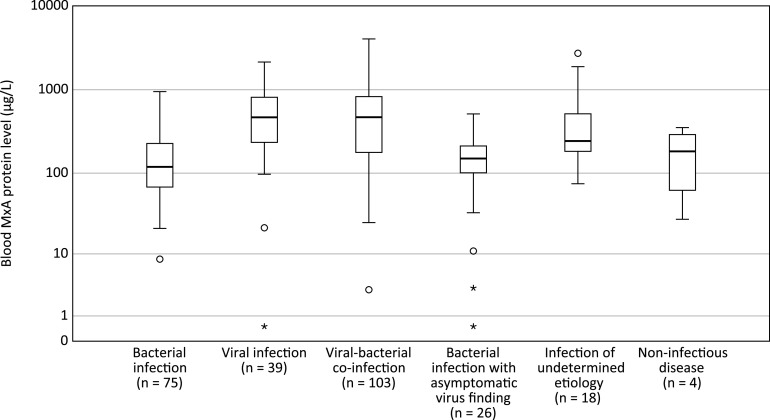
Blood MxA protein levels in 265 children hospitalized with an acute infection according to the etiology. For each group, the horizontal line represents the median, the box the upper and lower quartiles, and the whiskers the 95% confidence interval (CI). Circles indicate outliers extending beyond 1.5 times and up to three times the interquartile range, and asterisks indicate extreme values beyond three times the interquartile range. For pairwise comparisons of the groups “viral infection” and “viral-bacterial coinfection” with “bacterial infection” and “bacterial infection with coincidental virus finding,” *P < *0.001 for all comparisons (as determined by the Mann-Whitney U test).

The blood MxA levels (median [IQR]) were higher in children with a viral infection (467 [235 to 812] μg/L) compared to children with bacterial infection (119 [68 to 227] μg/L, *P < *0.001) or children with a bacterial infection and coincidental virus finding (150 [101 to 212] μg/L, *P < *0.001) (Fig. 2). In children with a viral-bacterial coinfection, the blood MxA protein levels were similar (469 [178 to 827 μg/L]) to children with a viral infection only (*P = *0.99).

**FIG 3:**
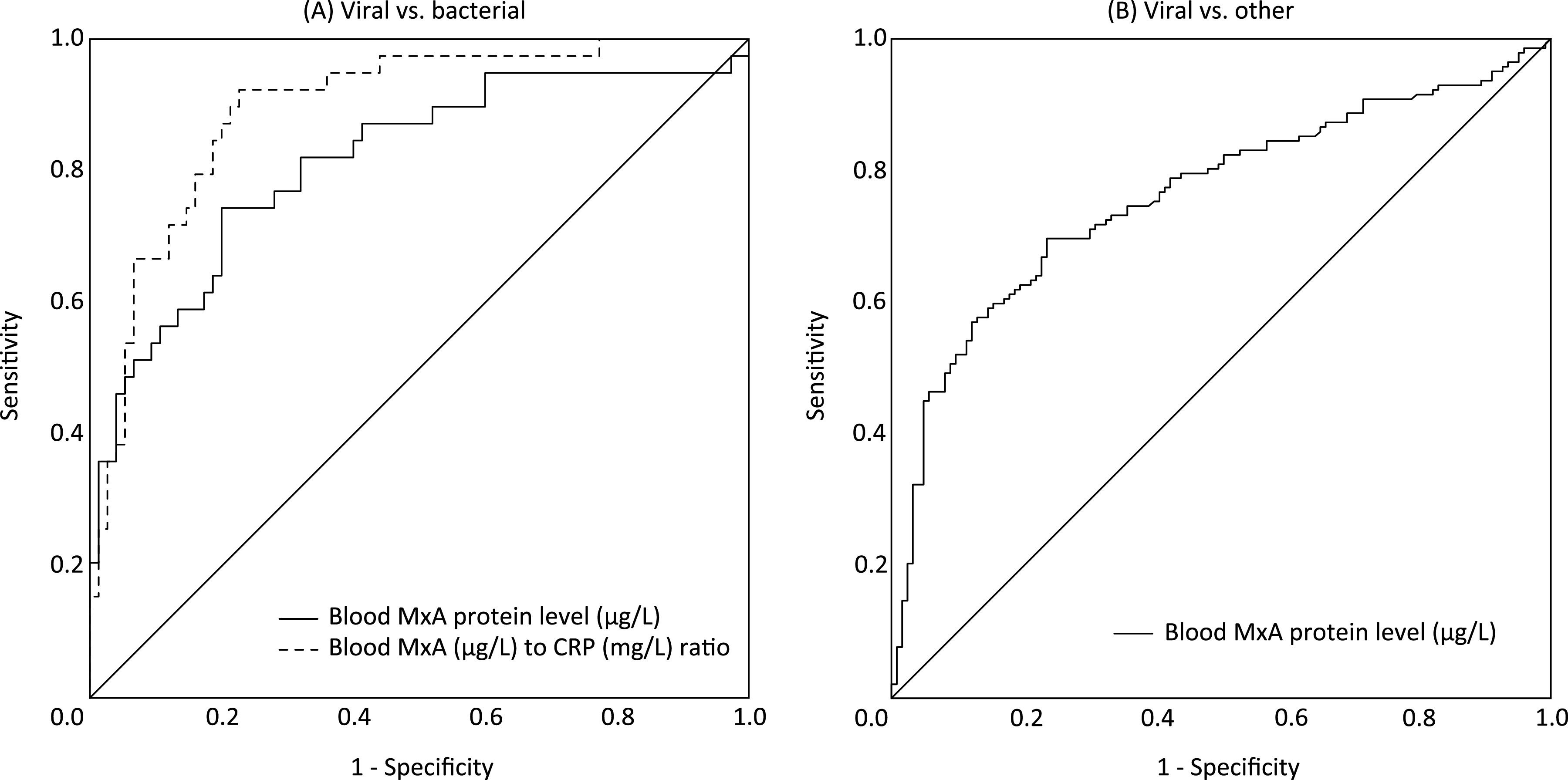
Differentiation between viral and bacterial infections by MxA and MxA/CRP ratio. (A) Receiver operating characteristic (ROC) curves for blood MxA protein level and blood MxA (μg/L) to CRP (mg/L) ratio in differentiating between children with a viral (*n* = 39) or bacterial (*n* = 75) infection. Area under the curve (AUC) = 0.81 (95% CI = 0.73 to 0.90) and 0.89 (95% CI = 0.83 to 0.96), respectively. (B) ROC curve for blood MxA protein level in differentiating between children (*n* = 142) with a symptomatic viral infection with or without a simultaneous bacterial infection and children (*n* = 105) without a symptomatic viral infection. AUC, 0.79 (95% CI = 0.73 to 0.85).

In a receiver operating characteristic (ROC) analysis for differentiation between viral (*n* = 39) and bacterial (*n* = 75) infections, blood MxA protein resulted in the area under the curve (AUC) of 0.81 (95% CI = 0.73 to 0.90) (Fig. 3A). The greatest sum of sensitivity (74.4%) and specificity (80.0%) for viral infections was obtained with a cutoff level of 256 μg/L.

We performed another ROC analysis to estimate the ability of blood MxA protein level to differentiate children (*n* = 142) with a symptomatic viral infection with or without simultaneous bacterial infection from children (*n* = 105) without a symptomatic viral infection (Fig. 3B). The AUC was 0.79 (95% CI = 0.73 to 0.85), and a cutoff level of 256 μg/L gave a sensitivity of 69.7% and a specificity of 79.0%.

The proportion of children with blood MxA protein level over the cutoff (256 μg/L) in each etiologic group are shown in Table S4 in the supplemental material.

### MxA to CRP ratio in differentiation between viral and bacterial infections.

In an ROC analysis, the blood MxA (μg/L) to CRP (mg/L) ratio gave the AUC of 0.89 (95% CI = 0.83 to 0.96) for differentiation between viral and bacterial infections (Fig. 3A). The greatest sum of a sensitivity (92.6%) and a specificity (77.3%) for viral infections were obtained with a cutoff level of 18.6. A cutoff level of 4.9 gave a sensitivity of 71.8% and a specificity of 88.0%.

### Viruses, MxA levels, and MxA/CRP ratio in children with bacteremic infections.

Blood bacterial culture was performed for 251 children, and it was positive for pathogenic bacteria in 13 (5.2%). Six of these children had a symptomatic, virus-positive respiratory infection and three were positive for a respiratory virus without respiratory symptoms. The median (IQR) blood MxA levels of children with a bacteremic infection with (*n* = 6) or without (*n* = 7) respiratory symptoms were 282 (127 to 535) μg/L and 122 (72 to 164) μg/L, respectively (*P = *0.18). Regardless of the presence of respiratory symptoms, all 13 children with a bacteremic infection had a MxA/CRP ratio below the cutoff value of 18.6, and all those without respiratory symptoms had a blood MxA level below the cutoff value of 256 μg/L.

### Effects of age on blood MxA levels.

**FIG 4:**
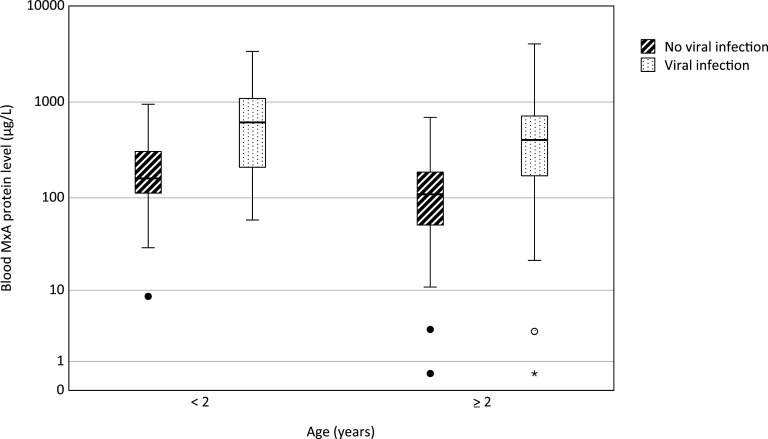
Effect of age on blood MxA protein levels in children without a symptomatic viral infection and with a symptomatic viral infection. For each group, the horizontal line represents the median, the box the upper and lower quartiles, and the whiskers the 95% CI. Circles indicate outliers extending beyond 1.5 times and up to three times the interquartile range, and asterisk indicates an extreme value beyond three times the interquartile range. For comparison of children <2 years and ≥2 years without a symptomatic viral infection, *P = *0.003, and for comparison of children <2 years and ≥2 years with a symptomatic viral infection, *P = *0.045 (as determined by the Mann-Whitney U test).

We first examined the effect of age on blood MxA protein concentration in children without a symptomatic viral infection. The median (IQR) MxA level was higher (160 [112 to 306] μg/L) in children (*n* = 39) less than 2 years of age compared to children (*n* = 66) at or above this age (109 [52 to 186], *P = *0.003) (Fig. 4). Thereafter, we examined the effect of age on MxA responses. In children with a symptomatic viral infection (with or without a bacterial infection), the median (IQR) blood MxA level was higher (614 [209 to 1,090] μg/L) in those<2 years of age (*n* = 50) compared to older children (403 [170 to 716], *n* = 92; *P = *0.045). In an ROC analysis in children <2 years of age, the AUC was 0.80 (95% CI = 0.70 to 0.89) for differentiation of children (*n* = 50) with a symptomatic viral infection with or without simultaneous bacterial infection from those (*n* = 39) without a symptomatic viral infection. A sensitivity of 70.0% and specificity of 76.9% were obtained with a cutoff level of 316 μg/L, whereas cutoff level of 524 μg/L gave the greatest sum of sensitivity (58.0%) and specificity (97.4%). In an ROC analysis in children aged ≥2 years, the corresponding AUC was 0.79 (95% CI = 0.72 to 0.86), giving a sensitivity of 67.4%, and a specificity of 86.4% with a cutoff level of 255 μg/L.

### Effects of live vaccines on blood MxA levels.

Thirty-two (12.0%) children had received a live attenuated virus vaccine within 30 days prior the enrollment (26, rotavirus; 3, measles, mumps, and rubella; and 3, varicella vaccine). We examined the effect of live vaccines on blood MxA protein levels in children <2 years of age who did not have a symptomatic viral infection. There was no substantial difference in the median (IQR) MxA levels between children (*n* = 14) recently vaccinated with a live virus vaccine (186 [150 to 321] μg/L) and children (*n* = 25) without a preceding vaccination (143 [99 to 282] μg/L, *P = *0.26).

### Sensitivity analysis.

We performed a sensitivity analysis restricted to children enrolled based on a suspected serious bacterial infection (*n* = 251). Characteristics of these children and those enrolled by suspected viral infection are shown in Table S3 in the supplemental material. The results remained essentially similar to the main analysis (see Fig. S1 and 2).

## DISCUSSION

We consistently found increased blood MxA protein levels in children with symptomatic viral infections, which were often accompanied by bacterial coinfections. We observed generally low MxA levels in children with bacterial infections regardless of coincidental respiratory virus findings in part of them. Our patient population consisted mostly of children presenting at the ED with a suspected serious bacterial infection; still, a respiratory virus was detected in 61% of children, and 39% were classified as having a viral-bacterial coinfection. Our results document the performance of blood MxA protein as a biomarker for symptomatic viral infection in children presenting with suspected serious infection and emphasize the high prevalence of viral-bacterial coinfections in such patients.

Earlier pathophysiologic and clinical studies indicate the importance of viral-bacterial coinfections in children (23–26). To our knowledge, this is the largest study of antiviral MxA responses in hospitalized children, which has included coinfections in the analyses. In previous studies, coinfected children have been lacking (19, 27, 28) or excluded (18) and often only children with definite viral or bacterial etiology have been compared. We included children with definite, probable, and mixed etiologies in our analyses. The pragmatic study design makes our results robust, although it probably decreased the observed sensitivities and specificities of MxA because of heterogeneity of groups.

Unfortunately, there is neither international standard, nor consensus reference range for blood MxA protein level. Few studies have reported MxA levels in asymptomatic children. Toivonen et al. (21) and Engelmann et al. (18) previously reported the median (IQR) blood MxA levels to be 110 (60 to 185) μg/L in 77 asymptomatic children aged 2 or 13 months, and 26 (3 to 75) μg/L in 44 uninfected children with a median age of 8 years, respectively. In the study of Nakabayashi et al., the mean MxA level was 77 (standard deviation, 62) μg/L in 52 healthy children with a mean age of 3.8 years (19). The normal range of blood MxA according to age should be better established in a larger cohort.

In parallel to our earlier finding of low MxA levels in healthy children with an asymptomatic respiratory virus infection (21), we report here low MxA levels in children with coincidental respiratory virus detection during bacterial infection. These results are consistent with a previous study reporting that asymptomatic rhinovirus infection did not induce a significant systemic transcriptional response in children, whereas symptomatic rhinovirus infection did (29). The advantage of measuring antiviral host response using MxA protein or other methods, such as gene expression profiling, is the ability to differentiate between incidental and pathogenic virus findings, which is not possible when detecting viruses by PCR. In addition, not all viruses are included even in the most comprehensive multiplex-PCR panels, and the turnaround times of PCR tests are variable, which may limit their usefulness in the initial diagnostic workup at the ED. MxA determination could be used in combination with direct detection of virus by PCR. Blood MxA protein measurement is technically straightforward and suitable for the development of rapid detection methods.

In our study, a blood MxA protein cutoff level of 256 μg/L best differentiated between viral and bacterial infections. Previous studies have reported slightly lower thresholds (18, 21). Engelmann et al. determined a cutoff of 200 μg/L by comparing children with respiratory syncytial virus, rotavirus, or other viral infections to healthy children. Of note, their study children with a confirmed viral infection were younger (median age, 0.6 years) than healthy controls (median age, 8 years). As we found higher baseline levels and stronger antiviral MxA responses in younger children, it might be reasonable to define age-stratified cutoff levels.

Given the high prevalence of viral-bacterial coinfections in children hospitalized with an acute infection, blood MxA protein probably cannot be used as a sole biomarker in such settings. This is due to the findings of similar MxA levels in children with viral infection only and viral-bacterial coinfection. The benefit of combined use of MxA and CRP in differentiating between viral and bacterial respiratory infections has been demonstrated, but mainly in adults and in outpatients (27, 28, 30). In our study, the blood MxA/CRP ratio yielded better sum of sensitivity and specificity than MxA measurement alone in differentiating between viral and bacterial infections. It should be noted here that attending physicians who set the diagnoses received the routinely measured CRP results but not MxA results.

We were able to recruit almost one-half of eligible children at the study EDs, and we included children with comorbidities. Thus, our study population should mimic the true patient population rather well. Another strength of the study was the systematic respiratory virus PCR testing. As the number of children with a viral infection without bacterial infection was lower than expected among those with blood bacterial culture drawn, we recruited an additional sample of children with suspected viral infections. Despite this, the study population as a whole was representative of suspected serious infections, and the sensitivity analysis excluding children recruited based on suspected viral infection supported our findings. A limitation of our study was the heterogeneous population with various infections which caused uncertainty in the etiological classification but, at the same time, it represents the real-world patient population. We considered a pragmatic study design to be essential since, in clinical practice, the challenge does not lie in differentiating asymptomatic children from those with infection of microbiologically confirmed etiology but in determining the cause of febrile illness as viral or bacterial in a timely and accurate manner at the ED.

We found blood MxA protein to be a promising biomarker for symptomatic viral infections in children with suspected severe infection. Viral-bacterial coinfections were frequently detected in our study, which needs consideration when novel biomarkers and diagnostic processes are developed. Blood MxA protein, potentially in combination with a biomarker for bacterial infections, should be further studied with the goal of improved targeting of antimicrobial treatments in febrile children.

## MATERIALS AND METHODS

### Study design and conduct.

This prospective diagnostic two-center study was conducted at the pediatric EDs of Turku University Hospital and Seinäjoki Central Hospital, Finland, between December 2016 and April 2018. The inclusion criteria for children with a clinical suspicion of serious bacterial infection were (i) age between 4 weeks and 16 years, (ii) admission to hospital, and (iii) blood bacterial culture drawn by the decision of the attending clinician. From June 2017 to April 2018, to ensure balance between viral and bacterial infections in the study population, we recruited a convenience sample of children with a suspected viral infection with the following inclusion criteria: (i) age between 4 weeks and 16 years, (ii) admission to hospital for an acute infection, (iii) no antibiotic treatment started on admission or used within 1 week prior to hospitalization, and (iv) venous blood samples (or insertion of an intravenous cannula) needed for other reasons. An exclusion criterion for all children was cancer under active treatment. Data on symptoms and recently administered vaccines were collected by parent-filled structured questionnaires and from the electronic registries of well-baby clinics. Blood and nasopharyngeal samples were collected upon admission at the ED from 261 children and within 48 h after admission from four children.

The study protocol was approved by the Ethics Committee of the Hospital District of Southwest Finland. The parents of all children, and older children or adolescents themselves, provided their written informed consent at the enrollment.

### Biomarker measurements.

Blood samples were collected by venous puncture. Blood bacterial culture, WBC count, and determinations of CRP and PCT levels in plasma were performed by routine methods in the hospital central laboratories. Whole-blood samples for MxA protein measurement were diluted 1:20 in hypotonic buffer and stored at −70°C until the enzyme immunoassay analysis was performed as described earlier (21).

### Virus detection.

Nasopharyngeal swab samples were suspended into phosphate-buffered saline, and nucleic acids were extracted using NucliSENS easyMag (bioMérieux). Multiplex RT-PCR Allplex respiratory panels 1 to 3 (Seegene) were used in the Turku University Hospital and FilmArray (BioFire Diagnostics) in Seinäjoki Central Hospital for the detection of respiratory viruses. Both methods detected adenovirus; influenza A and B viruses; parainfluenza viruses type 1, 2, 3, and 4; respiratory syncytial virus; human metapneumovirus; coronaviruses 229E, NL63, and OC43; rhinovirus; and enteroviruses. Allplex also detected human bocavirus and FilmArray coronavirus HKU1. FilmArray results for rhinovirus/enterovirus were further analyzed with Allplex to specifically document rhinovirus or enterovirus.

### Other diagnostic measures.

Other microbiological samples (e.g., urine sample or cerebrospinal fluid sample) were collected and radiographic imaging performed if needed to reach a specific diagnosis by the decision of the attending clinician.

### Classification of children according to etiology.

Etiologic groups and their definitions were decided *a priori.* Clinical diagnoses recorded at discharge by the attending clinician formed the basis for the classification. The diagnoses were verified by review of all clinical, laboratory, and radiologic imaging data from the electronic medical records. If there was any inconsistency regarding the diagnostic decision making, the final diagnosis was based on the expert opinion of two study physicians with expertise in pediatric infectious diseases who were blinded to MxA results. In cases where plasma C-reactive protein (CRP) or procalcitonin (PCT) values were utilized in the diagnostics, cutoff levels were 40 mg/L for CRP and 0.5 μg/L for PCT.

Children were classified into eight etiologic groups. A “definite bacterial infection” was defined as (i) a clinical diagnosis of sepsis, bacterial meningitis, bacterial type pneumonia (dense infiltration in X-ray and high CRP), pyelonephritis, septic arthritis, osteomyelitis, or other focal pyogenic infection; (ii) identification of pathogenic bacteria from a normally sterile site, or Mycoplasma pneumoniae or Chlamydia pneumoniae by PCR in a nasopharyngeal specimen; and (iii) no clinical or microbiological diagnosis of viral infection. A “definite viral infection” was defined as (i) a clinical diagnosis of upper respiratory tract infection, stomatitis, pharyngitis, tonsillitis, laryngitis, bronchiolitis, wheezy bronchitis, asthma exacerbation during viral respiratory infection, influenza, chickenpox, enteroviral disease, viral meningitis, or other clinically defined viral infection, and (ii) microbiologically confirmed viral etiology matching the clinical syndrome and no indication of bacterial etiology in microbiologic, hematologic, chemistry, or radiologic studies. A “probable bacterial infection” and “probable viral infection” were based on the same criteria as for definite infections but without microbiological confirmation. A “viral-bacterial coinfection” was defined as an infection with viral and bacterial etiology, or simultaneous viral and bacterial infections at distinct foci. A “bacterial infection with coincidental virus finding” was defined as definite or probable bacterial infection and a respiratory virus finding without respiratory symptoms or an incidental finding of another virus not causing symptoms. Children with an infectious disease that could not be categorized according to the above-mentioned criteria were classified as having “an infection of undetermined etiology.” A diagnosis other than an infectious disease was classified as “a noninfectious illness.”

For the analyses, we combined children with a definite or probable bacterial infection into a group of “bacterial infection” and children with a definite or probable viral infection into a group of “viral infection.”

### Statistical analyses.

We estimated that a sample size of 200 with the prevalence of 15% of both definite bacterial and definite viral infections would be sufficient for the planned comparisons. During the study we observed a lower rate of definite viral infections than anticipated and therefore recruited children with a suspected viral infection to ensure inclusion of at least 30 subjects in both these groups.

We compared blood MxA protein levels between above-defined etiologic groups using the Kruskal-Wallis test, followed by a pairwise Mann-Whitney U test. *P* values were adjusted for multiple comparisons by using the Bonferroni correction. ROC analysis was used to evaluate the capability of blood MxA protein level and blood MxA/CRP ratio to differentiate between patient groups. Cutoff levels were calculated from the ROC analyses using Youden index (sensitivity + specificity −1). We also present alternative cutoffs selected by potential clinical applicability. A sensitivity analysis was performed by excluding children enrolled based on suspected viral infection. Two-tailed *P* values of <0.05 were considered statistically significant. Statistical analyses were performed using SPSS, version 27.0 (IBM).
