# Supplementary material for: Myxovirus Resistance Protein A as a Marker of Viral Cause of Illness in Children Hospitalized with an Acute Infection
Source: Microbiol Spectr. 2022 Jan 26;10(1):e02031-21. doi: 10.1128/spectrum.02031-21 (PMC8791186; doi:10.1128/spectrum.02031-21)
Supplement: SUPPLEMENTAL FILE 1 — Supplemental material. Download SPECTRUM02031-21_Supp_1_seq6.pdf, PDF file, 0.2 MB [file spectrum02031-21_supp_1_seq6.pdf]

Supplementary Material

Piri R, Yahya M, Ivaska L, Toivonen L, Lempainen J, Nuolivirta K, Tripathi L, Waris M, Peltola V. Myxovirus Resistance Protein A as a Marker of Viral Cause of Illness in Children Hospitalized with an Acute Infection.

Supplementary Table 1. Age Distribution of 265 Study Children According to the Etiology 2

Supplementary Table 2. Clinical Diagnoses of 265 Study Children in Six Etiologic Groups 3

Supplementary Table 3. Clinical Characteristics, Diagnoses, and Detected Viruses and Bacteria in 251 Children with Suspected Bacterial Infection (Blood Bacterial Culture Obtained) and in 14 Children with Suspected Viral Infection 4

Supplementary Table 4. Proportion of Children with Blood MxA Protein Level Over the Cutoff of 256 µg/L in Six Etiologic Groups 6

Supplementary Figure 1. Blood MxA Protein Levels in 251 Children Hospitalized with Suspected Bacterial Infection (Blood Bacterial Culture Obtained) According to the Etiology 7

Supplementary Figure 2. Differentiation between Viral and Bacterial Infections by MxA and MxA to CRP Ratio in Children with Suspected Bacterial Infection (Blood Bacterial Culture Obtained)........ 8

Supplementary Table 1. Age Distribution of 265 Study Children According to the Etiology

Etiologic group	No. (%) of children	Median (IQR) age, years
Bacterial infection (definite or probable)	75 (28.3)	6.4 (1.3–9.1)
Definite bacterial infection	42 (15.8)	5.3 (0.4–8.2)
Probable bacterial infection	33 (12.5)	7.6 (2.9–10.3)
Viral infection (definite or probable)	39 (14.7)	6.3 (0.6–11.4)
Definite viral infection	31 (11.7)	6.3 (0.5–11.8)
Probable viral infection	8 (3.0)	6.2 (2.5–13.8)
Viral-bacterial co-infection	103 (38.9)	3.1 (1.5–5.3)
Bacterial infection with coincidental virus finding	26 (9.8)	2.6 (0.3–8.2)
Infection of undetermined etiology	18 (6.8)	3.3 (1.9–6.2)
Non-infectious disease	4 (1.5)	3.9 (0.7–8.8)

Abbreviation: IQR, interquartile range.

Supplementary Table 2. Clinical Diagnoses of 265 Study Children in Six Etiologic Groups

**Clinical diagnoses**	**No. (%) of children[a]**
Bacterial infection (n = 75)	
Pyelonephritis	28 (37.3)
Pneumonia	18 (24.0)
Skin or soft tissue infection	18 (24.0)
Group A streptococcal tonsillitis	3 (4.0)
Osteomyelitis	3 (4.0)
Sepsis	2 (2.7)
Other	3 (4.0)
Viral infection (n = 39)	
Viral respiratory infection[b]	20 (51.3)
Chickenpox, mononucleosis, or enteroviral disease	5 (12.8)
Gastroenteritis	4 (10.3)
Central nervous system infection	4 (10.3)
Other	6 (15.4)
Viral-bacterial co-infection (n = 103)	
Bacterial-type pneumonia with virus finding	63 (61.2)
Pyelonephritis with viral respiratory infection	11 (10.7)
Skin or soft tissue infection with viral respiratory infection	11 (10.7)
Viral respiratory infection with otitis media	4 (3.9)
Viral-bacterial tonsillitis	3 (2.9)
Gastroenteritis (viral-bacterial or viral with bacterial complication)	3 (2.9)
Chickenpox, mononucleosis, or enteroviral disease with bacterial complication	3 (2.9)
Viral encephalitis with bacterial complication	2 (1.9)
Other	3 (2.9)
Bacterial infection with coincidental virus finding (n = 26)	
Pyelonephritis	10 (38.5)
Sepsis or toxic shock syndrome	7 (26.9)
Skin or soft tissue infection	4 (15.4)
Central nervous system infection	2 (7.7)
Other	3 (11.5)
Infectious disease of undetermined etiology (n = 18)	
Tonsillitis of undetermined etiology[c]	10 (55.6)
Other infectious disease	8 (44.4)
Non-infectious disease (n = 4)	
Henoch-Schonlein purpura	2 (50.0)
Kawasaki disease	1 (25.0)
Sickle cell crisis	1 (25.0)

[a] Percentages were calculated within each etiologic group.
[b] Upper respiratory infection, wheezy bronchitis, laryngitis, tonsillitis, or influenza.
[c] Suspected bacterial tonsillitis with CRP > 40 mg/L but negative for group A streptococcus.

Supplementary Table 3. Clinical Characteristics, Diagnoses, and Detected Viruses and Bacteria in 251 Children with Suspected Bacterial Infection (Blood Bacterial Culture Obtained) and in 14 Children with Suspected Viral Infection

**Characteristic, diagnosis, or microbe**	**No. (%) of children**	
	**Suspected bacterial infection (n = 251)**	**Suspected viral infection (n = 14)**
Age		
1–2 months	27 (10.8)	1 (7.1)
3–11 months	27 (10.8)	2 (14.3)
1–2 years	62 (24.7)	3 (21.4)
3–6 years	58 (23.1)	3 (21.4)
7–15 years	77 (30.7)	5 (35.7)
Sex		
Female	129 (51.4)	10 (71.4)
Male	122 (48.6)	4 (28.6)
Chronic conditions		
None	190 (75.7)	9 (64.3)
Immunosuppressive disease or medication[a]	7 (2.8)	0 (0.0)
Other condition[b]	54 (21.5)	5 (35.7)
Disease characteristics		
Febrile (≥ 38.0 °C) before admission	222 (88.4)	8 (57.1)
Antibiotic treatment during hospitalization	232 (92.4)	2 (14.3)
Admitted to intensive-care unit	15 (6.0)	0 (0.0)
Clinical diagnoses		
Pneumonia	81 (32.3)	0 (0.0)
Pyelonephritis	49 (19.5)	0 (0.0)
Skin or soft tissue infection	33 (13.1)	0 (0.0)
Viral respiratory infection[c]	13 (5.2)	9 (64.3)
Tonsillitis	19 (7.6)	0 (0.0)
Sepsis or toxic shock syndrome	12 (4.8)	0 (0.0)
Central nervous system infection	8 (3.2)	0 (0.0)
Chickenpox, herpes zoster, mononucleosis, or enteroviral disease	8 (3.2)	0 (0.0)
Gastroenteritis	3 (1.2)	4 (28.6)
Osteomyelitis	5 (2.0)	0 (0.0)
Virus infection of undetermined etiology	7 (2.8)	1 (7.1)
Infectious disease of other or undetermined etiology	9 (3.6)	0 (0.0)
Non-infectious disease[d]	4 (1.6)	0 (0.0)
Respiratory viruses[e]		
Rhinovirus	73 (30.9)	5 (41.7)
Respiratory syncytial virus A or B	25 (10.6)	2 (16.7)
Human bocavirus	20 (8.5)	0 (0.0)
Adenovirus	12 (5.1)	3 (25.0)
Human metapneumovirus	12 (5.1)	0 (0.0)
Parainfluenza virus 1, 2, 3 or 4	10 (4.2)	0 (0.0)
Influenza virus A or B	9 (3.8)	2 (16.7)
Coronavirus V229E, NL63, OC43 or HKU1	11 (4.7)	0 (0.0)
Enterovirus	5 (2.1)	0 (0.0)
Other viruses		
Herpesviruses[f]	10 (4.0)	0 (0.0)
Rotavirus	1 (0.4)	2 (14.3)
Bacterial species isolated from blood or other sterile site[g]		
Streptococcus pneumoniae	6 (2.7)	0 (0.0)

Staphylococcus aureus	6 (2.7)	0 (0.0)
Escherichia coli	2 (0.8)	0 (0.0)
Haemophilus influenzae	2 (0.8)	0 (0.0)
Streptococcus intermedius	1 (0.4)	0 (0.0)
Salmonella paratyphi	1 (0.4)	0 (0.0)

[a] Juvenile arthritis with immunosuppressive medication (n = 2), severe combined immunodeficiency, cartilage-hair hypoplasia, liver transplant, sickle cell disease, or total lectin pathway deficiency (n = 1 for each).
[b] Urological or renal disorder (n = 12), neurological disorder or syndrome (n = 12), asthma (n = 10), gastrointestinal disorder (n = 5), cardiovascular disease (n = 4), endocrine disorder (n = 4), hematologic disorder (n = 3), premature birth at < 32 weeks (n = 3), other (n = 6).
[c] Upper respiratory infection, wheezy bronchitis, laryngitis, or influenza, with or without otitis media or other localized bacterial complication.
[d] Henoch-Schonlein purpura, Kawasaki disease, or sickle cell crisis.
[e] Percentages were calculated of children studied for respiratory viruses by multiplex PCR (n = 236 for suspected bacterial infection and n =12 for suspected viral infection).
[f] Herpes simplex virus, varicella-zoster virus, Epstein-Barr virus, or human herpesvirus 7.
[g] Cerebrospinal fluid, pleural fluid, or lymph node biopsy.

Supplementary Table 4. Proportion of Children with Blood MxA Protein Level Over the Cutoff of 256 µg/L in Six Etiologic Groups

**Etiologic group**	**No. (%) of children with MxA >256 µg/L**
Bacterial infection (n = 75)	15 (20.0)
Viral infection (n = 39)	29 (74.4)
Viral-bacterial co-infection (n = 103)	70 (68.0)
Bacterial infection with coincidental virus finding (n = 26)	6 (23.1)
Infection of undetermined etiology (n = 18)	7 (38.9)
Non-infectious disease (n = 4)	1 (25.0)

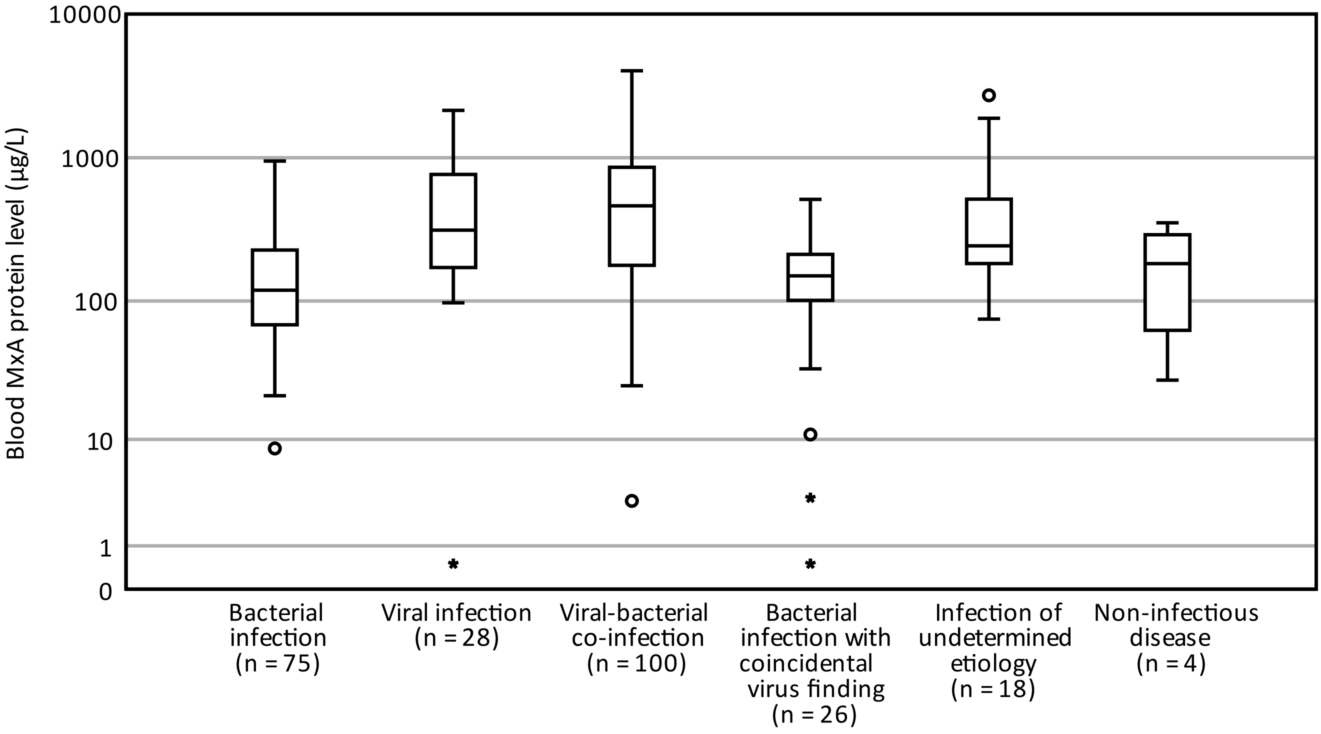

Supplementary Figure 1. Blood MxA protein levels in 251 children hospitalized with suspected bacterial infection (blood bacterial culture obtained) according to the etiology. For each group, the horizontal line represents the median, the box the upper and lower quartiles, and the whiskers the 95% confidence interval. Circles indicate outliers extending beyond 1.5 times and up to 3 times the interquartile range, and asterisks indicate extreme values beyond 3 times the interquartile range. For pairwise comparisons of group ”Viral infection” with ”Bacterial infection” and ”Bacterial infection with coincidental virus finding”, $P < .001$ and $P = 0.01$, respectively, and of group ”Viral-bacterial co-infection” with ”Bacterial infection” and ”Bacterial infection with coincidental virus finding”, $P < 0.001$ for both comparisons, by Mann-Whitney U test.

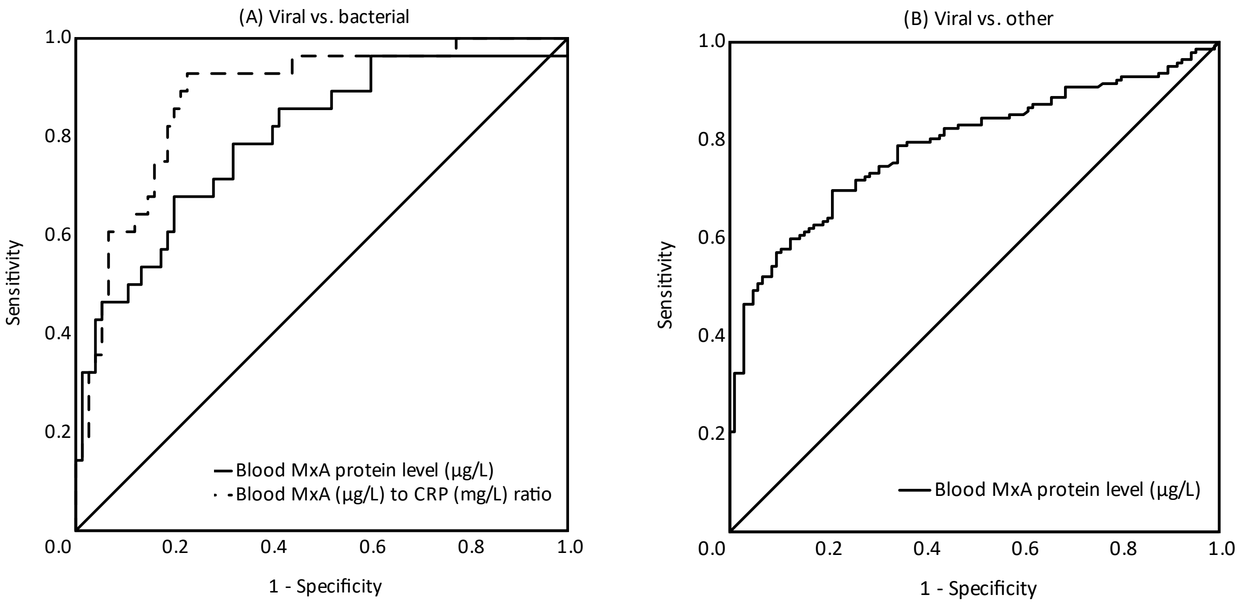

Supplementary Figure 2. Differentiation between viral and bacterial infections by MxA and MxA to CRP ratio in children with suspected bacterial infection (blood bacterial culture obtained). (A) Receiver operating characteristic (ROC) curves for blood MxA protein level and blood MxA (µg/L) to CRP (mg/L) ratio in differentiating between children with a viral (n = 28) or bacterial (n = 75) infection. Area under the curve (AUC), 0.80 (95% CI, 0.70–0.90) and 0.88 (95% CI, 0.81–0.95), respectively. (B) ROC curve for blood MxA protein level in differentiating between children (n = 128) with a symptomatic viral infection with or without a simultaneous bacterial infection and children (n = 105) without a symptomatic viral infection. AUC, 0.78 (95% CI 0.73–0.84).
